# Potency of Pfeiffer’s Crystallization to Analyze Oral Leukoplakia and Squamous Cell Carcinoma

**DOI:** 10.31557/APJCP.2020.21.2.517

**Published:** 2020

**Authors:** Vanshika Makkar, Mala Kamboj, Anjali Narwal, Rajeev Kumar Kapoor

**Affiliations:** 1 *Oral and Maxillofacial Pathology and Oral Microbiology, Post Graduate Institute of Dental Sciences, *; 2 *Department of Microbiology, Maharishi Dayanand University, Rohtak, Haryana, India. *

**Keywords:** Crystallization test, diagnostic test, oral Leukoplakia, oral Squamous cell Carcinoma and Transverse form

## Abstract

**Objective:**

Oral cancer usually has an early precancerous stage before its actual malignant transformation. Although there are various approaches to diagnose early stages of cancer, yet there is one less explored, cost effective and simple technique known as crystallization test. The aim of the study was to reaffirm the effectiveness of Pfeiffer’s crystallization test in screening oral leukoplakia and squamous cell carcinoma.

**Methods:**

Fifty oral leukoplakia, sixty five oral squamous cell carcinoma and sixty healthy individuals participated in crystallization test. Single blood drop was used to perform the test and obtained crystal patterns were analysed. Cross tabulation and Chi-Square test was performed to find the frequency and association between the groups. Kruskal-Wallis H test and Mann Whitney U test was applied comparing mean transverse form.

**Results:**

Sensitivity of crystallization test was 80% and 93.84% in oral leukoplakia and squamous cell carcinoma. Chi-Square analysis revealed highly significant transverse form between the study groups (p < 0.000).

**Conclusion:**

Crystallization test proves to be simple, reliable and minimal invasive diagnostic approach under strictly maintained physical conditions.

## Introduction

Oral squamous cell carcinoma (OSCC) is the most common malignant neoplasm of oral cavity, which arises from oral epithelium. In India over one lakh new oral cancer cases are registered every year (Warnakulasuriya, 2009). Transformation of normal oral epithelial tissue to invasive squamous cell carcinoma (SCC) emulates multiple paths and has multifactorial etiology, with an intermediate stage of precancer (Kuffer and Lombardi, 2002).

Inceptive lesions of oral cancer manifest as small, localized erosion or erythema, which could remain painless until the lesion becomes ulcerated or increases in size affecting routine function. Majority of the cases remain undiagnosed until they attain a visibly detectable size (Silverman, 1988), Sarode et al., (2013). Mortality of cancer can dwindle if the lesions are perceived, diagnosed and treated at an early stage. So, there is a pressing need for a simple, non-painful method of early cancer detection. 

Various diagnostic approaches including cytological, biochemical, biopsy, light based and optical diagnostic methods have been evolved over a period of time. Adjunct to these there is one interesting physical test, Crystallization technique that is simple, quick, cost effective, reliable and quite confirmatory (Sarode et al., 2013; Shaikh et al., 2012). Pfeiffer pioneered it in the year 1938, in regard to the diagnosis of tuberculosis in cattle and he further extended this study to the detection of human cancer. According to him under certain experimental conditions, the process of crystal formation is sensitive to inclusion of small quantities of foreign substances derived from living organisms. Addition of diluted hemolysed human blood to Cupric Chloride (CuCl_2_) solution when allowed for crystallization unveiled surprising differences in the form, depending upon whether the blood is originated from healthy or unhealthy persons (Sabarth and Williams, 1975).

The present study was conducted to detect crystallization test (Ct) in Oral Leukoplakia (OL) and to equate it with OSCC and normal blood. The Transverse form (TF) patterns observed were defined and classified, so that something conceptual is promulgated for future studies.

## Materials and Methods

*Selection and Description of Participants*

Present diagnostic, cross-sectional and case control study was conducted in the Department of Oral Pathology and Microbiology, PGIDS, Rohtak, Haryana, India. Blood samples were taken from clinically diagnosed and graded cases of OL, OSCC and healthy controls to observe the crystal pattern formation in Ct.

OL was clinically grouped into four types (WHO, 1998) namely early or thin, homogenous or thick, granular or verruciform and speckled or erythroleukoplakia. Clinically ulceroproliferative lesions with indurated margins were included in OSCC group and graded according to TNM staging. Controls were age and sex matched, without any apparent oral lesions and who gave no history of any systemic disease or deleterious habits. 

A total of 197 subjects participated in the study, out of which 22 cases without confirmatory histopathological diagnosis were dropped out (10 in group II and 12 in group III). So the final number evaluated was 175. Study groups comprised of group I (control) 60, group II (OL) 50 and group III (OSCC) 65 participants. In Group II and III clinical diagnosis was further confirmed by histopathological report. Participants with prior history of radiotherapy or chemotherapy and those giving any history of systemic illness were excluded. 

*Technical information*

A 20% working solution of Copper (II) chloride, (CuCl_2_) anhydrous, 99%, obtained from ACROS Organics™ was prepared and filtered before use. Under aseptic conditions, by pricking ring finger with a lancet one drop of blood was collected. Using micropipette 50μl of blood was collected and was mixed with 1cc of double distilled water and hemolysed blood sample was prepared. 0.2cc of this was added to 10cc of 20% cupric chloride solution. The mixture was gently poured into a flat bottomed, pre warmed petri dish of 4-inch diameter and was placed undisturbed in a BOD incubator at 30^o^C and (35% - 55% humidity) for 18 hrs. 20% CuCl_2_ solution alone served as control. 

*Assessment*

Crystal patterns obtained in the petri dishes were studied using hand lens against broad daylight. Three independent observers evaluated the crystal pattern, out of which two were blinded for case and control to eliminate any bias. Plates were observed for the presence, number and appearance of transverse forms. Color and texture of crystals formed, number and location of centers of nucleation were observed.

*Ethics*

The study was initiated after obtaining clearance from the Institutional Ethics Committee (Ethical Approval No-PGIDS/IEC/2016/97). 

*Statistics*

Data was analyzed using Statistical Package for Social Sciences (SPSS) software Version 25.0 for inferential analysis. Sensitivity, specificity, positive and negative predictive values of the Ct were tabulated. Cross tabulation and Chi-Square test was performed to find the frequency and association between the groups. Mean TF was calculated for all the groups and data was checked for normality (Shapiro-Wilk test). Data analyzed was non-parametric so Kruskal-Wallis H test was applied for comparing more than 2 groups and Mann Whitney U test for comparison among 2 groups. 

## Results

Demographic data of participants enrolled in study revealed age range from 21 to 76 years with a mean age of 50.25 years in group I, 43.16 in group II and 56.43 in group III. Male predominance was seen in all the three groups with 77.71% males and 22.28% female participants. Most common site of presentation in group II was bilateral retro commissural area (58%) and among group III it was retromolar trigone region (21.53%).

**Figure 1:**
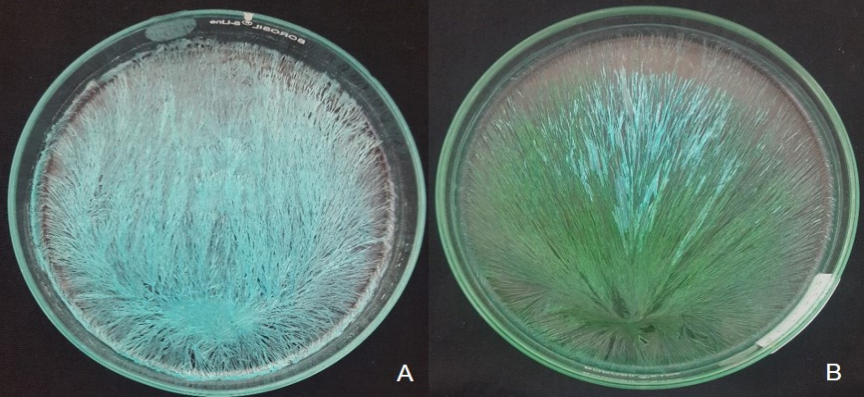
A, Cupric Chloride Crystals Alone; B, Crystallization Pattern of Normal Blood

In the present study crystallization patterns were observed in 175 subjects. Control CuCl_2_ solution showed blue crystals, which were arbitrarily arranged at varying angles with multiple centers of nucleation and secondary, tertiary branches. Sabarth and Williams, 1975 described similar patterns as Messy or “Muddle pattern” (Figure 1A).

**Figure 2:**
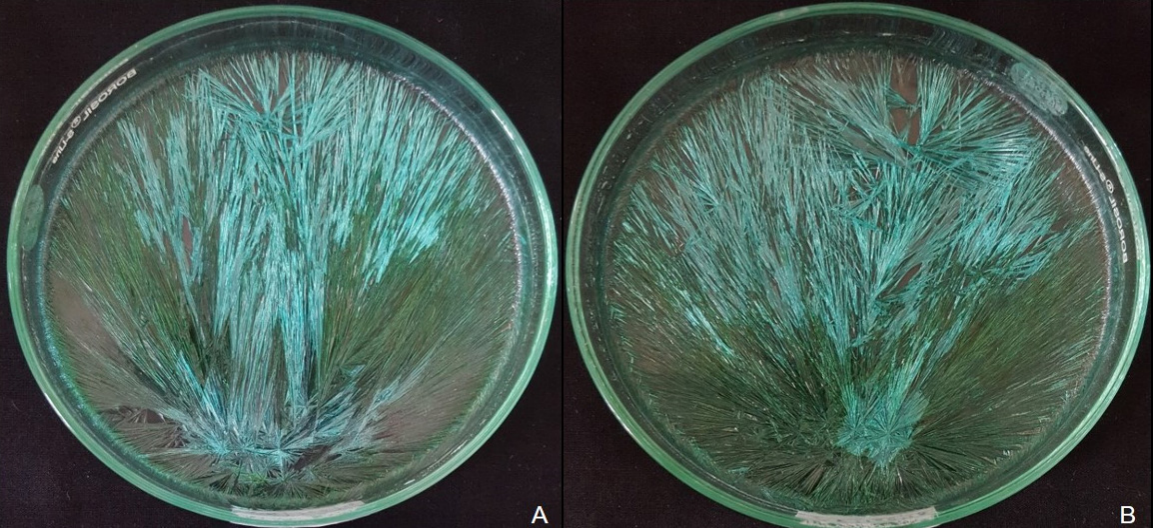
A, Crystallization Pattern Observed in OL; B, Crystallization Pattern Observed in OSCC

Addition of hemolyzed blood originating from healthy individual to CuCl_2_ solution influenced the pattern of crystallization and produced long and short radiating crystals originating from eccentrically located center of gravity (Figure 1B). Any disturbance in this pattern accorded the impression of a disease state in the individual (Figure 2 A, B)

Formation of crystals showed peculiar arrangement in CuCl_2_ solution, which was considered as TF. Appearance of TFs was further categorized into - horizontal bar, star shaped (multi-directional fanning) or unidirectional fanning. 

**Figure 3:**
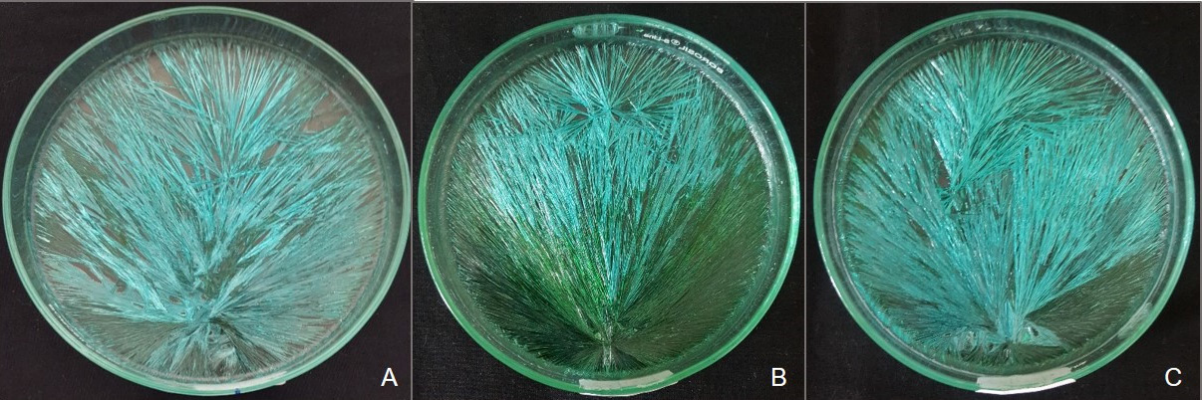
Various Transverse form Patterns A, Horizontal Bar; B, Star; C, Fanning

Horizontal bar recorded in present study was a sharp straight line that can either be short or long. Though majority of the continuous radiating crystals failed to cross this line, few impinged it at varying angles (Figure 3a) Star type of TF was considered as a single point from which crystals radiated in all the directions. This point was other than the eccentric COG (Figure 3b). Fanning was read as a change in the orientation of crystals radiating in a unidirectional manner (Figure 3c). Hollow spaces were recorded as empty spaces of convex lenses of variable sizes, which were seen around these TFs. 

Presence of TF as horizontal bar, star or fanning was considered as a positive test. Ct was affirmative in 61 out of 65 cases of group OSCC, (sensitivity was 93.84%). In group II, Ct was positive in 40 out of 50 cases (80% sensitivity). 43 cases of group I were negative for Ct and 17 (28.33%) showed false positivity for the test. Thus specificity of Ct was 71.66%. Positive and negative predictive values of the Ct in OL were computed as 70.17% and 81.13% and OSCC 78.20% and 91.48% respectively Table 1.

The total number of TFs were counted which stood highest in group III (60.42%), followed by group II (30.08%) and group I (8.55%). Mean of total TFs also escalated from group I to III (0.483±0.85 < 2.04±1.3 < 3.07±1.75) and mean TFs were significantly higher in oral cancer cases as compared to the other groups (p value 0.000) Table 2. Dividing the TFs into horizontal bar, star and fanning between the three groups, all the forms were significantly higher in Group III > II> I. (p value 0.000). 

Correlation of test results with clinical and histopathological classification of group II is represented in the Table 3. 

Based upon clinical appearance, Group III cases were staged according to TNM staging system (Table 3) and were graded into well differentiated (WDSCC) and moderately differentiated (MDSCC) upon their histopathological diagnosis. The comparison between TNM staging of group III and various TF patterns did not reach up to the level of significance. Out of 65 cases in group III hollow spaces were present in 30. Hollow spaces were highest in stage III (68.75%) > stage II (50.0%) > stage I (40%) > stage IV (15.38%). Inter-group comparison revealed significantly higher hollow spaces in stage III (p value 0.036). 

In WDSCC and MDSCC, positive outcome was seen in 92.68% and 95.83% cases respectively. Number of star formation was significantly increased in MDSCC (p value 0.003). 

Present study also revealed variations in the center of gravity (COG) as one, two or diffuse centers, more than one COG was found in 28.57% cases. However these variations in center patterns did not reach up to the level of significance between the groups. The radiating pattern was also noted from COG that depicted single winged radiating pattern which predominated among all the groups, followed by two and three winged. Two-winged pattern was statistically significant in group II (p value 0.031). Three-winged pattern was highest among the cases of group III (p value 0.03). 

**Table 1:** Results of Crystallization Test

Crystallization Test Results	Diagnosis			Total
	Diseased		Non-Diseased	
	III* (n=65)	II* (n=50)	I (n=60)	(n=175)
Positive	61	40	17	118
Negative	4	10	43	57
Sensitivity	93.84%	80%	-	-
False Negative	6.15%	20%	-	-
Specificity	-	-	71.66%	-
False Positive	-	-	28.33%	-

* Biopsy Proven Cases; Positive predictive value (PPV) and Negative predictive value (NPV) of Crystallization test in detecting OL is 70.17% and 81.13% respectively; PPV and NPV of Ct in detecting OSCC is 78.20% and 91.48% respectively.

**Table 2:** Comparison of Mean and Total Transverse Forms among the Groups*

Group	Transverse Form			No. of Total TF	Mean TF ± SD
	Horizontal Bar	Star	Fanning		
I	17	3	9	29 (8.55%)	0.483±0.85
II	18	44	40	102 (30.08%)	2.04±1.3
III	81	75	52	208 (60.42%)	3.07±1.75

H value – 74.474, df = 2, p value – 0.000

**Table 3:** Details of Study Participants in Group II and Group III

Clinical Appearance Group II				
	Thin smooth	Thick fissured	Granular verruciform	Erythro-leukoplakia
No. of Cases	3	33	1	13
Positive crystallization	3	24	1	12
Histopathological Diagnosis Group II				
	Hyperkeratosis with no dysplasia	Mild dysplasia	Moderate dysplasia	Severe dysplasia
No of case	3	34	12	1
Positive crystallization	2	26	11	1
Clinical Staging Group III				
				
	TNM Stage I	TNM Stage II	TNM Stage III	TNM Stage IV
No. of cases	10	26	16	13
Positive crystallization	9	25	15	12
Histopathological Diagnosis Group III				
				
	Well differentiated SCC		Moderately differentiated SCC	
No. of cases	41		24	
Positive crystallization	38 (92.68%)		23 (95.83%)	

## Discussion

Oral cancer is a debilitating disease with increasing incidence worldwide (Warnakulasuriya and Cain, 2011). It is a complex disease possessing multifactorial etiology, which acts through multitudinous pathways upon normal oral epithelial tissue leading to its transformation into cancer (Warnakulasuriya, 2009). 

Male predominance was strikingly higher in both OL (98%) and OSCC (83.07%) in the present study. Remarkable difference in the gender could be due to the fact that intake of tobacco in any form between the age of 15-49 years is more among males (57%) than females (11%) (Manoharan and Tyagi, 2010). Site variation among the subjects may be linked to the form of tobacco intake. Cawson and Odell, (1998) reported that anterior buccal mucosa was the most commonly affected site in bidi smokers whereas posterior mucosa was associated with chewing habits. Regarding OSCC, (Rhodus, 2005) narrated that in Indian population buccal mucosa was the most commonly affected site, where as in present study retro molar trigone was most predominant.

Any alteration in the human body could be linked to modulated biochemical levels in the body fluids. In malignancy various enzymes are involved in nucleic acid metabolism and the destruction of surrounding tissue. They release breakdown products within the blood circulation that could be responsible for disease specific pattern in Ct (Sarode et al., 2013; Burkhardt,1985). The formative forces present in blood provide an orientation to the CuCl_2_ solution, creating a definitive pattern of crystallization (Sabarth and Williams, 1975).

E Pfeiffer in 1938 worked for the very first time on CuCl_2_ crystallization and observed star formation in inflammation, and TF in cancer (Gruner, 1940). Basic principle proposed behind the test was that the colloidal proteins that are present in extremely dilute solution in human blood act as impurities when mixed with CuCl_2_ solution (Gulati et al., 1994). Another possible rationale could be the presence of increased concentration of protein degraded products such as polyamines and diamines in the blood of diseased (Sarode et al., 2013; Shibata, 1994). 

Kopaczewski (1933), stated that during evaporation, molecules move at different rates and amplitudes (Gruner, 1940). Cimpean C was of the opinion that small amount of additive in CuCl_2_ solution acts the same way as iron dust if placed in magnetic field, gets arranged along the lines of magnetic field (Cimpean and Hotiu, 2014). Morris and Morris, (1939), (1941) conducted a work on solution of sugar, glycine, glycogen, certain cereal grain extracts and some seed extract and noticed varied pattern formation. He proposed that variation in surface tension might affect crystallization.

The crystals that appear in the test are physical ones as no new chemical substance is formed (Gruner, 1940). Though crystallization is the property of inorganic salts, it has been observed that magnesium sulfate and lead acetate have also been used but CuCl_2_ crystallization is most sensitive (Sabarth and Williams, 1975, Gulati et al., 1994). The impetus could be that copper is present as various coenzymes in our body and chloride is also present in the blood as an osmoregulator (Gulati et al., 1994). 

Sensitivity of Ct for Group II came out to be 80% in the present study. Tarigoppula et al., (2018) conducted a study on 30 premalignant lesions and sensitivity was 83.3%. One more study on 50 cases of OL by Rawat et al., (2018) reported absence of transverse forms in all the cases and presence of leaf like pattern in 92% cases of oral leukoplakia Shaikh et al., (2012) evaluated 39 cases of precancer on female genital tract and sensitivity was 84.15%. Outcome of present study was in concordance with the studies of Tarigoppula et al., (2018) and Shaikh et al., (2012). However the appearance of leaf form defined by Rawat et al., (2018) could be correlated with the fanning TF observed in our study. 

As pre-cancer is a stepping-stone to develop oral cancer, so the embarking of TFs in premalignancy probably indicates the magnitude of PMDs that could transform into malignancy. Subjects with increased number of TFs must be kept under regular follow-up as they might show malignant changes over a period of time. 

Sensitivity of the present study (93.84%) is also in accordance with findings of previous studies on oral cancer by Sarode et al.,( 2013) (96%) and Tarigoppula et al., (2018) (96.3%). Outcome of similar studies on different body sites are also congruent with the present study namely Quadeer (1980) (94.15%), Shaikh et al., (2012) (94.6%), Gruner (1940) (90.1%), and Gulati et al., 1994 (88%).

The specificity in the present study was 71.66% which is low as compared to previous studies of Quadeer (1980), Gruner (1940), Sarode et al., (2013), Gulati et al., (1994) and Shaikh et al., (2012). This difference could be due to the fact that controls were not screened for the presence of any systemic disease using biochemical and serological tests, only history was recorded. 

Sarode et al., (2013) reported the positive and negative predictive values (PPV and NPV) of the test in detecting OSCC as 97.96% and 93.55% respectively. Tarigoppula et al., (2018) stated PPV and NPV of oral cancer was 83.87% and 97.06%, and for oral PMDs it was 83.33% and 86.84% respectively. In present study PPV was comparatively lower (70.17%) due to false positivity in control group. 

Among histopathological grades of OSCC mean TF was higher in MDSCC as compared to in WDSCC similar to the findings stated by Tarigoppula et al., (2018). However, the results were not statistically significant in both the studies. Whereas akin comparison by Sarode SC et al., (2013) was statistically significant. This difference could probably be attributed to the lower number of MDSCC cases recorded in the present study. 

Comparison between clinical stages of OSCC in this study revealed highest mean TF in TNM stage III (3.25±1.69) > stage II (3.23±1.69) > stage I (2.80±1.98) > stage IV (2.76±1.48). Sarode et al., (2013) obtained similar correlation where the frequency was highest among stage II (5.8±4.658%) > stage III (5.75±2.417%) > stage IV (4.90±2.315%). In both the studies difference was not statistically significant. A third study by Gulati et al., (1994) also observed the maximum number of TFs in stage III and IV but no such correlation was seen between the stages of carcinoma and number of TFs. Probable reason could be due to regional lymph node metastasis that occurs till stage III so body responses are more enhanced at that time. 

Comparison of hollow spaces with clinical staging of OSCC was significantly higher in TNM stage III. Presence of hollow spaces signifies the gravity of disease therefore could be considered as good indicator of correlating with clinical stages of the disease.

Upon histopathological examination in OL group, Ct showed higher reliability for detecting moderate (91.66%) than mild dysplasia (76.47%). Study with a larger sample size could confirm the correlation among grades of dysplasia and presence of TFs.

Out of the subtypes of TFs, though all forms were specific for the disease but presence of horizontal bar outshined in cases of malignancy although star and fanning were ineffectual for differentiating PMDs and malignancy. 

All the plates were evaluated between 18 hours of crystal formation. Color of crystals was variable and showed changes from green to blue upon constant evaporation. The texture of crystals was also variable from fine to coarse. Some variations from the normal pattern were also evaluated which includes appearance of more than one COG and more than one radiating patterns from COG. 

Center denotes the initial point of crystallization. Different centers may either be present around the main center or diffusely seen over the whole surface. Sabarth and Williams, 1975 have also quoted similar findings and described them as variations of normal pattern in healthy as well as diseased group. 

Another finding was radiating patterns of crystals from the COG. Majority of the cases (62.28%) had single radiating wing like structures indicating that all the crystals radiated from a single point of COG. Two-winged pattern was significantly higher in group II whereas three-winged pattern was higher in group III. It could be inferred that as the magnitude of the disease elevated, the number of radiating patterns also increased. Similar radiating patterns were also observed and described by Sabarth and Williams, 1975 as variations of Ct. No such disease correlation was cited in their study.

*Strength of the study*

Minimal invasive and cost effective technique for screening. Present study appears to be a first attempt to correlate Ct of OL with clinical classification and histopathological grades of dysplasia. Its use could effectively be extended to detect oral PMDs. Prospective studies with larger sample size could further endorse this fact. 

*Limitations*

Ct is greatly influenced by temperature, atmospheric humidity and vibrations during the process of evaporation and working conditions of the incubator. Any mold or dust particles could influence the results. Test is purely physical one, so controlling factors are of utmost importance for the accuracy of results. 

In conclusion, Ct proves to be a simple, cost-effective supplemental diagnostic test under strict physical conditions. It effectively detects OL and OSCC. Prospective studies with larger sample size could further endorse this fact. Ct for future studies could be to detection of malignancy in inaccessible areas and to analyze the response to treatment therapy. By means of this physical test it can be inferred that every drop of blood indicates the complete bodily condition along with the pathological state of a person.
